# Blood pressure response to combined general anaesthesia/interscalene brachial plexus block for outpatient shoulder arthroscopy

**DOI:** 10.1186/1471-2253-14-50

**Published:** 2014-06-30

**Authors:** Hauke Janssen, Roland von Stosch, Rupert Pöschl, Benedikt Büttner, Martin Bauer, José Maria Hinz, Ingo Bergmann

**Affiliations:** 1 Department of Anaesthesiology, Emergency and Intensive Care Medicine, University of Göttingen Medical School, Robert-Koch Str. 40, Göttingen 37075, Germany; 2 Orthopaedic Clinic for Outpatient Surgery, Baunatal, Germany; 3 Anaesthesia Clinic for Outpatient Surgery, Baunatal, Germany

**Keywords:** Bezold-Jarisch reflex, Interscalene brachial plexus, Outpatient surgery, Regional anaesthesia, Shoulder surgery

## Abstract

**Background:**

Shoulder surgery is often performed in the beach-chair position, a position associated with arterial hypotension and subsequent risk of cerebral ischaemia. It can be performed under general anaesthesia or with an interscalene brachial plexus block, each of which has specific advantages but also specific negative effects on blood pressure control. It would be worthwhile to combine the advantages of the two, but the effects of the combination on the circulation are not well investigated. We studied blood pressure, heart rate, and incidence of adverse circulatory events in patients undergoing shoulder surgery in general anaesthesia with or without an interscalene block.

**Methods:**

Prospective, randomised, blinded study in outpatients (age 18 to 80 years) undergoing shoulder arthroscopy. General anaesthesia was with propofol/opioid, interscalene block with 40 ml 1% mepivacaine. Hypotension requiring treatment was defined as a mean arterial pressure <60 mmHg or a systolic pressure <80% of baseline; relevant bradycardia was a heart rate <50 bpm with a decrease in blood pressure.

**Results:**

Forty-two patients had general anaesthesia alone, 41 had general anaesthesia plus interscalene block. The average systolic blood pressure under anaesthesia in the beach-chair position was 114 ± 7.3 vs. 116 ± 8.3 mmHg (p = 0.09; all comparisons General vs. General-Regional). The incidence of a mean arterial pressure under 60 mmHg or a decrease in systolic pressure of more than 20% from baseline was 64% vs. 76% (p = 0.45). The number of patients with a heart rate lower than 50 and a concomitant blood pressure decrease was 8 vs. 5 (p = 0.30).

**Conclusion:**

One can safely combine interscalene block with general anaesthesia for surgery in the beach-chair position in ASA I and II patients.

**Clinical trial number:**

DRKS00005295.

## Background

Shoulder surgery is often performed with the patient in a semi-reclining position with the upper body elevated (“beach-chair”). There is a high incidence of hypotension in this position, which is the cause for some concern due to the risk of insufficient cerebral perfusion [1-3].

The anaesthetic technique is either general anaesthesia or regional block, usually an interscalene brachial plexus block. A combination would give the greater intraoperative patient comfort of the general and the superior postoperative pain relief of the regional anaesthetic [4,5]. Blood pressure decreases during general anaesthesia as a result of several factors [6]. The average blood pressure reduction seen under interscalene block is generally less pronounced, but there are sudden episodes of severe hypotension with bradycardia caused by the Bezold-Jarisch reflex [1,7,8].

Haemodynamic stability has been studied individually for each technique as well as for the combination of the two [7,9-11], but with the exception of one small study [9], there is no systematic investigation comparing the incidence and severity of hypotensive events under general anaesthesia alone and general anaesthesia plus interscalene block.

We studied the perioperative course of blood pressure and heart rate in patients undergoing shoulder arthroscopy in the beach-chair position under general anaesthesia alone or combined with an interscalene block. Bispectral index monitoring was used to minimize the risk of awareness while avoiding excessive propofol plasma concentrations.

It was our hypothesis that the additional interscalene block would not increase the incidence of severe hypotensive and/or bradycardic events.

## Methods

This prospective, randomised, double-blinded comparative study was conducted with the approval of our institutional review board (Ethikkommission der Universitätsmedizin Göttingen) and is registered in the German Clinical Trials Register (DRKS00005295). We recruited ASA grade I and II patients 18 to 80 years of age scheduled for outpatient shoulder arthroscopy in an orthopaedic outpatient clinic (Orthopädische Praxisklinik, Baunatal, Germany), who were scheduled for treatment by the same team of surgeon (JS) and anaesthetist (IB). The participating patients gave their written informed consent. Exclusion criteria were allergy to local anaesthetics and chronic pain patients with a treatment regimen of daily opioids and/or additional drugs such as gabapentin or pregabalin. Patients were excluded from analysis if the block was not successful, or if they required a conversion to an open procedure or were admitted postoperatively to hospital. The procedures were performed under either general anaesthesia alone or under general anaesthesia combined with an interscalene block (ISB). The investigator registering and analysing the perioperative data (HJ) was blinded to group allocation.

On arrival in the operating suite, standard monitoring consisting of ECG, non-invasive blood pressure, pulse oximetry, and bispectral monitoring (BIS) was started, and an intravenous infusion was established in a peripheral vein. Blood pressure was measured on the contralateral arm with a cuff chosen to match the circumference of the patient's arm. Midazolam was administered in a mildly sedative dose (1–3 mg IV).

The block was performed by two investigators (IB and RP). We used a nerve stimulator (Stimuplex® HNS 11, Braun Melsungen) to localise the interscalene brachial plexus. When the needle was correctly positioned with a motor response of the deltoid and/or triceps muscles to a 0.1 ms stimulus with a current less than 0.3 mA, we injected 40 ml of 1% mepivacaine without adrenaline. The extent of the block was tested by warm-cold and pinprick discrimination and change in muscle tone. The block was considered successful if discrimination and motor function were lost. We performed the block in a separate regional anaesthesia induction room. For blinding purposes all patients were routed through this room. The patient's neck was not visible to the recording investigator (HJ).

The general anaesthetic was a total intravenous technique with propofol and remifentanil or sufentanil. Remifentanil (0.5 μg kg^−1^) or sufentanil (0.2 - 0.3 μg kg^−1^) were given at induction. The maintenance remifentanil infusion rate started at 0.3 μg kg^−1^ min^−1^ and was adjusted up or down according to clinical indicators. Some patients were given sufentanil intraoperatively in bolus doses of 10 to 20 μg before increasing the remifentanil infusion rate. This was at the discretion of the anaesthetist. Propofol was initially infused at a rate of 1 mg kg^−1^ min^−1^ until the BIS index had decreased to below 60. The rate was then reduced to maintain the BIS index between 40 and 60 [12,13]. Orotracheal intubation was facilitated with mivacurium (0.2 mg kg^−1^).

Heart rate and peripheral oxygen saturation were monitored continuously, and arterial blood pressure was measured non-invasively at five-minute intervals. The obtained data were stored on-line in the monitoring system. The baseline values were recorded in the supine patient just before induction of general anaesthesia, *i.e.* after the interscalene block had taken effect. Severe hypotension was defined as a systolic blood pressure lower than 80% of baseline or a mean arterial pressure lower than 60 mmHg, and was treated with vasopressors (Akrinor®, a combination of cafedrine and theodrenaline) and additional intravenous fluids. Severe bradycardia was defined as a heart rate lower than 50 beats per minute and was treated with atropine if accompanied by a drop in blood pressure.

Nursing personnel blinded to group allocation observed the patients in the recovery room for approximately two hours postoperatively. The discharge criteria were stable vital signs, no nausea or vomiting, tolerable pain and a return of motor function in the patients with ISB. The patients were given a prescription for ibuprofen 600 mg three times daily and metamizole 1 g four times daily for the first two days. Tramadol 50 mg up to four times daily was prescribed as rescue medication for severe pain. Complications, such as Horner syndrome or dyspnoea, and their treatment were recorded in the chart.

On the second day after surgery, an investigator blinded to the group allocation contacted the patients by telephone at home and interviewed them with regard to pain on the standard numeric rating scale of 1 to 10, the amount and type of analgesics taken, the occurrence of nausea, vomiting or shivering, and the degree of satisfaction (NRS 1 to 6; very good to very poor) with the anaesthetic. With the exception of the process times, the data were analysed by one investigator (HJ), who was blinded to the group allocation of the patients.

### Statistical analysis

The primary outcome was the incidence of arterial hypotension requiring treatment. Secondary outcome variables were bradycardia, process times, and severity of postoperative pain. The data were analysed with the statistics program StatSoft® (StatSoft Inc. USA). Continuous data were tested for normal distribution with the Kolmogorov-Smirnov test. Normally distributed data were described with mean and standard deviation. Categorical data were given as percentages. Continuous and ordinally scaled data were compared with the Student t-test, categorical data with Fisher's exact test. A probability of error of less than 5% (p < 0.05) was defined as significant. We based our calculation of the necessary sample size on the blood pressure data reported by Choi et al. [10] for patients in general anaesthesia in the beach-chair position. They reported an average systolic blood pressure of 99.6 ± 18 mmHg in anaesthetised patients without interscalene block in the beach-chair position. We considered a 10% decrease in average blood pressure (to 90 mmHg) to be clinically relevant. Calculations showed that 32 patients per group would be required to detect a difference of this magnitude with a power of 0.80 and an alpha-error of less than 0.05 (http://www.statisticalsolutions.net/pss_calc.php). We recruited two groups of 42 patients each to compensate for potential dropouts.

## Results

**Figure 1:**
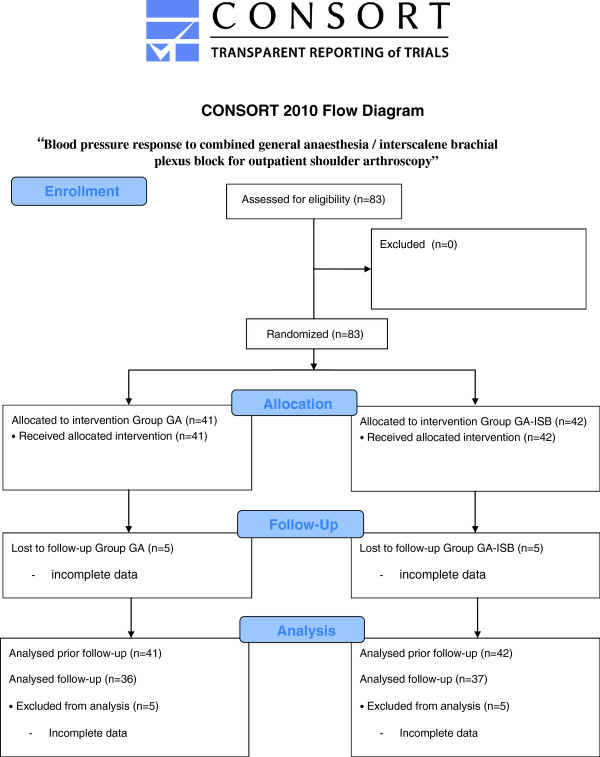
Consort flowchart.

Recruitment was closed after eighty-three patients had been included in the study. Forty-one had general anaesthesia (GA), and 42 had general anaesthesia and interscalene block (GA-ISB). Comparable numbers of patients had chronic beta-receptor blocker medication in each group. No patient had to be excluded after recruitment. Patient characteristics are given in Table 1, Figure 1.

**Table 1:** Patient characteristics

	**GA**	**GA + ISB**	**p**
	**(n = 42)**	**(n = 41)**	
Sex: male/female (n)	19/23	18/23	0.25
Age (years)	51 ± 10	53 ± 9	0.17
Height (cm)	170 ± 7	170 ± 8	0.25
Weight (kg)	80 ± 14	81 ± 16	0.60
ASA classification I/II (n)	11/31	16/25	0.35
Chronic beta-blocker therapy (n (in % of group))	7 (17)	5 (12)	0.56

(*Abbreviations*: GA General anaesthesia, GA + ISB General anaesthesia with interscalene brachial plexus block).

The block was successful in all patients, with loss of warm/cold and pinprick discrimination and motor function. No patient required a rescue block, a measure not provided for by the study protocol. An inadequate block would have led to the exclusion of the patient from the study, and the operation would have been performed without the block.

Table 2 shows the intraoperative courses of blood pressure and heart rate. Although baseline and overall average systolic blood pressures were the same in both groups, the maximum systolic pressure was higher, the minimum pressure was lower, and the decrease from baseline was greater in the group with additional ISB. The heart rate was consistently lower in the GA-ISB group.

**Table 2:** Hypotensive episodes, infused fluid volumes and circulatory parameters in the patients in the general anaesthesia group (GA) and the general anaesthesia with interscalene block group (GA-ISB)

	**GA**	**GA-ISB**	**p**
	**(n = 42)**	**(n = 41)**	
Hypotension requiring vasopressor treatment (n (%))	27 (64%)	31 (76%)	0.45
Bradycardia requiring atropine (n)	8	5	0.30
Infused fluid volumes (ml)	1488 ± 209	1536 ± 447	0.54
Systolic arterial blood pressure (mmHg)			
Supine before induction	132 ± 5.6	138 ± 17	0.11
After positioning in beach-chair			
Average	114 ± 7.3	116 ± 8.3	0.09
Maximum	135 ± 15	146 ± 19	< 0.005
Minimum	96 ± 11	90 ± 14	< 0.02
Maximum change from baseline (%)	−27%	−34%	< 0.002
Heart rate (beats per minute)			
Supine before induction	77.6 ± 11.6	70.0 ± 12.8	< 0.01
After positioning in beach-chair			
Average over anaesthesia time	73.2 ± 8.3	67.4 ± 10.4	< 0.01
Maximum	83.9 ± 9.1	77.9 ± 12.7	< 0.02
Minimum	66.3 ± 9.1	60.4 ± 9.3	< 0.02
Maximum change from baseline (%)	9.1 ± 10.8	13.8 ± 23.0	0.24

The numbers of patients requiring circulatory support with atropine or vasopressors and the infused fluid volumes were the same in both groups.

The propofol requirements were similar in both groups (Table 3). The opioid doses represent the total of induction and maintenance doses related to anaesthesia time. There was no difference in opioid consumption between the groups.

**Table 3:** Consumption of anaesthetic drugs (median (25th; 75th percentile)) in the patients in the general anaesthesia group (GA) and the general anaesthesia with interscalene block group (GA-ISB)

	**GA**	**GA-ISB**	**p**
	**(n = 42)**	**(n = 41)**	
Propofol (mg kg^−1^ h^−1^)	7.6 (6.2; 9.7)	7.7 (5.1; 9.8)	0.7
Remifentanil (n)	34	32	
Remifentanil (μg kg^−1^ min^−1^)	0.08 (0.05; 0.11)	0.08 (0.04; 0.12)	0.6
Sufentanil (n)	35	13	
Sufentanil (μg kg^−1^ h^−1^)	0.60 (0.49; 0.72)	0.60 (0.42; 0.73)	0.9

The “n” in parentheses refers to the number of patients in each group who was given the respective opioid. Most patients were given both.

No complications of the regional block, such as Horner syndrome or phrenic nerve paresis, were observed. Neither chest radiographs nor thoracic ultrasound were performed to definitively rule out phrenic nerve paresis, but no patient complained of dyspnoea, and no clinical signs of phrenic nerve paresis were seen or reported.

Table 4 shows the documented perioperative process times. The “anaesthesia preparation time” (arrival anaesthetist until patient cleared for surgery) was longer in GA-ISB group (p < 0.001), but the “ready for surgical preparation time” measured from the patients’ arrival in operating theatre until they were cleared for surgery, and during which anaesthesia blocks surgical use of the theatre, was shorter in the GA-ISB group. Surgical time was shorter in the GA-ISB group. There was no difference in theatre emergence time (end of surgery to patient leaving operating theatre). Total anaesthesia time was longer in the GA-ISB group.

**Table 4:** Process times

	**GA**	**GA-ISB**	**p**
	**(n = 42)**	**(n = 41)**	
ISB time	n.a. ‡	34.2 ± 19.0	n.a.
Ready for surgical preparation time	31.1 ± 13.4	18.1 ± 6.0	< 0.001
Surgical time	56.0 ± 12.4	46.0 ± 15.3	< 0.002
Dressing complete to PACU† time	11.1 ± 4.3	11.1 ± 3.6	0.71
Anaesthesia control time	42.2 ± 15.0	31.3 ± 8.3	< 0.001
Total anaesthesia time	42.2 ± 15.0	65.5 ± 21.8	< 0.001

*ISB = interscalene block; † PACU = post anaesthesia care unit; ‡ n.a. = not applicable.

“ISB* time”: From disinfection of the skin until block fully developed.

*“Ready for surgical preparation time”*: Time from arrival in the operating theatre until end of anaesthesia induction;

*“Surgical time”*: From incision to skin closure and dressing;

*“Theatre emergence time”*: From end of surgery until leaving the operating theatre;

*“Anaesthesia control time”*: Ready for surgical preparation time plus theatre emergence time;

*“Total anaesthesia time”*: ISB time plus anaesthesia control time;

*“PACU † time”*: From arrival in the PACU to the eligibility for discharge.

Equal numbers of patients in both groups were available for the postoperative interview (Table 5). The groups did not differ with regard to preoperative pain, postoperative analgesic consumption, postoperative nausea and vomiting or satisfaction with the anaesthetic. However, the patients with the ISB had less severe postoperative pain on the day of surgery and had a tendency to less pain on postoperative day one.

**Table 5:** Results of the patient interviews

	**GA**	**GA-ISB**	**p**
	**(n = 36)**	**(n = 37)**	
Preoperative pain			
Patients with preoperative pain (%)	97	100	0.31
Preoperative pain intensity (NRS*)	7.3 ± 2.0	7.4 ± 1.5	0.7
Patients taking analgesics (%)	64	64	0.93
Postoperative pain - incidence (%)			
Day of surgery	86	68	0.06
Post-op day 1	86	78	0.39
Post-op day 2	86	84	0.78
Postoperative pain intensity (NRS 1–10)			
Day of surgery	4.1 ± 2.6	2.7 ± 2.6	0.02
Post-op day 1	3.8 ± 2.4	2.7 ± 2.3	0.06
Post-op day 2	3.2 ± 2.3	2.7 ± 2.2	0.33
Postoperative analgesics required (% of patients)			
non-opiate analgesics	83	83	0.96
opiate analgesics	64	49	0.19
Postoperative complications - incidence (%)			
Nausea	22	11	0.19
Vomiting	19	11	0.30
Shivering	3	8	0.32
Paraesthesias persisting > two days	0	0	0.97
Success of operation			
Pain alleviated (%)	81	70	0.31
Mobility improved (%)	78	68	0.33
Satisfaction with anaesthetic (NRS 1–6)	1.5 ± 0.7	1.5 ± 0.9	0.75
Would recommend anaesthetic (%)	92	100	0.07

*NRS = Numeric rating scale.

NRS for pain: 1 = no pain to 10 = worst pain;

NRS for satisfaction: 1 = very satisfied to 6 = totally dissatisfie.

## Discussion

This is the first study comparing the blood pressure and heart rate changes in patients undergoing shoulder arthroscopy in the beach-chair position under general anaesthesia alone or under a combination of general anaesthesia and interscalene block. The results of this investigation showed that adding an interscalene brachial plexus block to a general anaesthetic did not increase the incidence of hypotensive or bradycardic episodes requiring therapeutic intervention.

The interscalene plexus was located by electrical stimulation. We did not use ultrasound, a very useful technique for this purpose, because we did not have the necessary equipment. However, despite its limitations, nerve stimulation is still a valid and accepted method for performing regional anaesthesia of peripheral nerves [14]. The technique is effective in experienced hands, and the block was successful in all of our patients.

There was a high incidence of low blood pressure requiring treatment with vasopressors in both groups, but the rates of 64% in the general anaesthesia group and 76% in the combination group are well within the published ranges. While the definitions of hypotension are not identical in the publications [2,7,11], they are similar enough to allow a comparison. Trentman et al. using criteria much more stringent than ours, reported a rate of hypotension from 54% to up to 75% in patients with chronic antihypertensive medication [7]. Kwak et al. [11], who used criteria similar to ours, reported an incidence of hypotension of 64%, while Yadeau et al. [2] observed hypotension with systolic blood pressure below 90 mmHg in virtually every patient of their study.

The maximum decrease in systolic blood pressure from baseline was significantly greater in the patients with interscalene block, but from a higher baseline, and the minimum values did not require therapy more often than in the group with only a general anaesthetic. We take this as evidence that the observed hypotension was related to setting the patient in the semi-upright position soon after the induction dose of propofol [6], since most treatment events were triggered by a decrease of systolic blood pressure after induction of anaesthesia. In a similar, smaller study on younger patients, in which all patients had an ISB while one group had an additional general anaesthetic, Ozzeybek et al. found that the combination of the two techniques caused a significantly greater decrease in arterial blood pressure than the ISB alone [9].

The heart rate was lower at all measuring points in the patients with an interscalene block. A similar percentage of patients in both groups had chronic medication with beta-adrenergic receptor blockers, and we suggest that the lower heart rate was due to the effects of the interscalene block, since it is known to induce a Bezold-Jarisch reflex with bradycardia [8]. Unfortunately we did not document the heart rate before establishing the block but only immediately prior to induction of general anaesthesia, a point in time at which the block had already taken effect. In a minority of patients, treatment was required for bradycardia complicated by hypotension, which we would consider to be a Bezold-Jarisch reflex event. This occurred in eight patients of the general anaesthesia group but in only five patients with the additional interscalene block, which argues against an increased risk of sudden hypotension and bradycardia with the combination.

While we did not expect the dose of propofol required to maintain BIS within the prescribed range to differ between the groups, but we had proposed that the administered doses of the opioid analgesic would be lower in the patients with the regional block. However, the latter was not the case, and we offer two possible explanations for this observation. The actually required maintenance doses of remifentanil or sufentanil may have been lower in the GA-ISB group, but the operations were relatively short, and the standardised high induction doses might have masked the difference. An alternative explanation is that the lack of a quantitative measure of analgesia may have led to unnecessarily high intraoperative opioid doses, since clinical signs of inadequate analgesia are not very reliable, and there are no clinical signs of more than adequate analgesia. Supplemental doses may have been administered more according to experience than to actual need, and one is more likely to increase than to reduce a standard infusion rate of remifentanil.

The time required for performing the ISB was similar to times described in the literature [15,16]. The duration of the operation was significantly shorter in the group with the ISB, which in is accordance with published reports [15,17,18]. The reason for this is thought to be a reduced amount of bleeding due to the lower blood pressures in the patients with the regional block.

“Anaesthesia control time”, the cumulative time needed for induction and emergence in the operating theatre, was significantly shorter by about 13 minutes in the group with the regional block, since all preparations and the block itself were performed in a separate room parallel to the on-going operation; only the induction was performed in the operating theatre. This allowed a significantly more rapid turnover and better theatre utilisation with the interscalene block. These results are similar to those published by D'Alessio et al. [15] and Gonano et al. [19]. On the other hand, “total anaesthesia time”, the time during which an anaesthetist is occupied with the patient, was significantly longer in the group with the ISB, since a second anaesthetist was required to perform the block parallel to the on-going operation. Gonano et al. [19] compared the economical aspects of general anaesthesia versus interscalene block and found that the overall anaesthesia costs were lower with the regional technique. Although their study only compared general anaesthesia with ISB alone, the data for personnel and operating theatre costs per minute given in their publication showed that the combined general and regional technique would still cost less than using general anaesthesia alone due to the more effective theatre utilisation despite the additional cost for the second anaesthetist.

Patient satisfaction was high in both anaesthesia groups, but the groups differed in pain severity. The patients in the GA-ISB group had significantly less pain on the evening after surgery. On post surgery day one the scores also differed with a p-value of 0.06, which indicates that the lower pain severity registered in the ISB group was probably not due to chance. Such a difference that persists past the duration of action of mepivacaine could be evidence of a pre-emptive effect of the regional anaesthesia [4]. Ozzeybek et al. [9] studied the course of postoperative pain severity in patients undergoing shoulder surgery under general anaesthesia with ISB. However, since the authors failed to give the actual pain scores, it is not possible to compare their results with those in our corresponding GA-ISB group. The comparison would have been difficult in any case, since their patients had an interscalene catheter and were managed with patient-controlled interscalene analgesia for the first 48 hours.

The interpretation of our data is limited to some extent by the fact that blood pressure and heart rate values were not recorded until after the interscalene block had taken effect, and also by the fact that the anaesthetists were allowed to use either remifentanil or sufentanil or a combination of the two. A further limitation was that it was impossible to totally blind the nursing staff to the group allocation; those patients with ISB were unable to move their arms as opposed to those without ISB. This may have influenced patient treatment and dispensing of analgesics in the postanaesthetic care unit, but this unavoidable “unblinding” would not have compromised either the primary cardiovascular outcome data or the pain scores, since the former were extracted from the anaesthesia chart and monitor, and the latter were obtained by telephone interview.

## Conclusions

An interscalene brachial plexus block can be safely used in ASA I and II patients in addition to general anaesthesia for shoulder surgery in the beach-chair position; the incidence of clinically relevant hypotension or of Bezold-Jarisch reflex events is not increased.

## Competing interests

The study was financed by departmental funds, including the purchase of all devices and materials used in the study. During the past five years none of the authors have received any form of reimbursement or financial or non-financial support from a company that could gain or lose financially from the publication of this manuscript. None of the authors hold any stocks or shares in a company that would gain or lose financially from the publication of this manuscript. None of the authors are applying for any patents related to the content of the manuscript. There are no other competing financial or non-financial interests.

## Authors’ contribution

IB, HJ and MB designed the study and interpreted the results. IB, RP and RS recruited and treated the patients, HJ analysed the stored data offline and performed the statistical analyses together with JMH and BB. All authors collaborated in discussing the results and drafting the manuscript. All authors read and approved the final manuscript.

## Pre-publication history

The pre-publication history for this paper can be accessed here:

http://www.biomedcentral.com/1471-2253/14/50/prepub
